# *TFR2* gene alterations in idiopathic erythrocytosis reinforce a possible relation between erythrocytosis and iron metabolism

**DOI:** 10.1016/j.gendis.2024.101291

**Published:** 2024-04-05

**Authors:** Andrea Benetti, Irene Bertozzi, Maria Luigia Randi

**Affiliations:** First Medical Clinic, Department of Medicine – DIMED, University of Padova, Padova 35128, Italy

At present, most cases of sporadic absolute erythrocytosis remain without an etiologic demonstration, excluding acquired secondary forms, polycythemia vera, and hereditary erythrocytosis. Therefore, about 70% of all patients with erythrocytosis, receive a diagnosis of idiopathic erythrocytosis (IE) meaning that physicians failed to recognize the cause of the hemoglobin and hematocrit increase.^1^

In clinical practice, a significant number of IE patients have high or borderline-high ferritin levels,^1^ suggesting that, at least in some cases, erythrocytosis coexists with an impaired iron balance. Hereditary hemochromatosis (HH) is a disease characterized by raised serum ferritin and iron overload in several organs: the type 1 form is due to alterations in *HFE* gene and it has been shown that *HFE* variants alter the supply of iron to the erythroid tissues even if changes in erythropoiesis in HH are not mediated by *HFE* expression in nucleated red cells.^2^ Other types of HH are due to alterations in other genes involved in iron metabolism such as hepcidin, ferroportin, hemojuvelin, and transferrin receptor 2.

Postulating that a possible underlying cause for erythrocytosis could be the impairment in iron metabolism, we documented yet the presence of *HFE* mutations^1^ in several IE patients, and now we describe variants in another gene involved in iron metabolism.

We evaluated 118 patients (male/female = 101/17; mean age = 53.7 ± 17.2 years) with IE, all coming from north-eastern Italy, diagnosed with the following criteria: i) hemoglobin >165 g/L and hematocrit >49% in males and hemoglobin >160 g/L and hematocrit >48% in females, (ii) long-standing unexplained erythrocytosis, (iii) no relatives with increased hemoglobin or hematocrit, (iv) no evidence of smoke, arterial-venous shunt, pulmonary and/or renal diseases or neoplasms, and (v) absence of somatic mutations in *JAK2* p.Val617Phe or variants in *JAK2* exon 12. Patients carrying hemoglobin variants with high oxygen affinity, as evaluated by venous p50, and those with a left-shifted oxygen dissociation curve were excluded. No children younger than 16 years of age are included in the present cohort being our surgery dedicated only to adults. All patients gave written informed consent. The protocol was approved by the local Institutional Ethical Committee (Azienda Ospedaliera di Padova, ref: 3922/AO/16). The study was conducted in compliance with the principles of the Declaration of Helsinki.

A targeted next-generation sequencing panel for patients with erythrocytosis, containing *HAMP*, *FTL*, *FTH*, *SLC11A2*, *SLC40A1*, *HFE*, *HFE2*, and *TFR2* genes, was set up to explore the presence of variants in iron metabolism genes. *HFE* variants (p.His63Asp in 41, p.Cys282Tyr in 10, p.Glu277Lys in 1, and p.Ser65Cys in 3 cases) were present in 51 patients (43.2%). Fourteen out of these 51 patients (27%) had also other genes' variants. *TFR2* variants were observed in 11 unrelated patients (9.3%) (Fig. 1A, B). Their main clinical and laboratory data are reported in Table S1. The predictable significance of *TFR2* variants is summarized in Table S2. Five out of the 11 patients (45%) had other genes' variants: 2 patients had *EGLN1* (p.Cys127Ser or p.Gln157His), 1 patient had *JAK2* (p.Leu113Val), 1 patient had *HFE* (p.His63Asp), and 1 patient had *JAK2* (p.Thr78Ile) alteration with also *HFE* (p.His63Asp/Cys282Tyr) variants. At present, to our best knowledge, the *TFR2* variants we found have never been related to erythropoiesis. It is interesting to note that in at least 4 of our patients, other genes' variants known to be involved in erythropoiesis (*EGLN1*C127S, *EGLN1*Q157H, *HFE*H63D, *HFE*H63D/C282Y, and *JAK2*T78I) were recognized. Unfortunately, the paucity of patients failed to give evidence of differences in hemoglobin and hematocrit levels comparing those with isolated *TFR2* variant and those with other genes' alterations. All variants reported are germline and no patients had *HAMP*, *FTL*, *FTH*, *SLC11A2*, *SLC40A1*, and *HFE2* variants.

**Figure 1:**
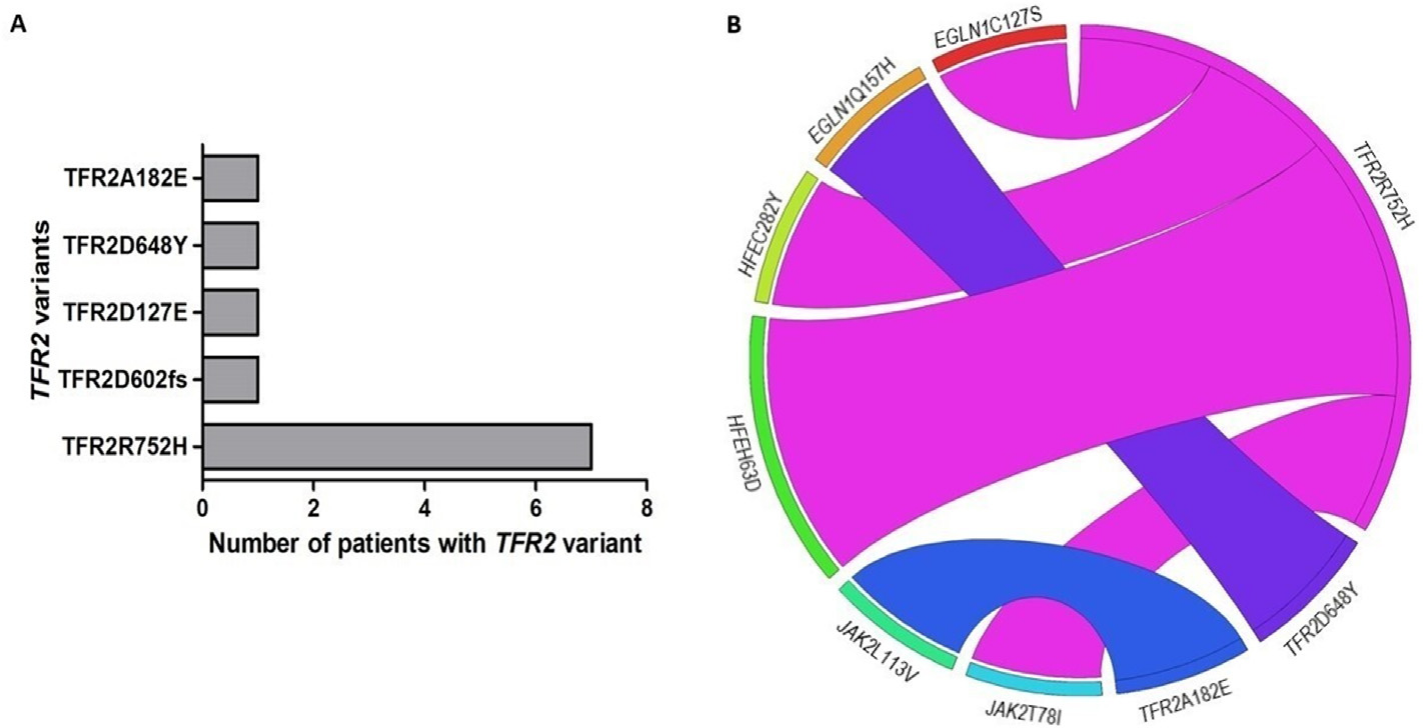
Distribution of TFR2 variants in the 11 patients. **(A)** Number of patients for each variant of *TFR2*. **(B)** Circos plot representing the correlation between *TFR2* variants and other variants in other genes.

Erythropoiesis in humans is the predominant consumer of iron, and we hypothesize that an impairment in iron metabolism could be a possible underlying cause of erythrocytosis. Ferritin concentration has a positive even if not significant correlation with hemoglobin production and its correlation with red blood cell count is different in subjects with elevated (negative) or normal (positive) iron concentrations. Erythrocytosis in symptomatic hemochromatosis is due to elevated transferrin saturation levels which results in increased iron uptake by erythroid precursor cells; iron is then utilized by these precursor cells to increase red blood cell synthesis.^2^^,^^3^

A recent paper^2^ showed that *HFE* p.His63Asp gene variant seems to increase iron concentration promoting red cell production and in heterozygous patients p.His63Asp, the most common variant of the *HFE* gene, an altered impact on erythropoiesis was found in the presence of increased iron content.

In 2018, we observed that *HFE* variants are significantly more common in patients with erythrocytosis^1^ compared with the European general population (13.6%), and in the present paper, we confirm this remark.

Transferrin receptor 2 (TFR2), homologous to TFR1, is a transmembrane protein expressed in the liver and in erythroid cells considered a sensor of circulating iron in a complex with erythropoietin receptor. This receptor appears to be critical for erythropoietin receptor transport to the cell surface and for terminal differentiation. Moreover, in animal models, TFR2 modulates the sensitivity of erythroid cells to erythropoietin and endogenous erythropoietin; in mice, the loss of *TFR2* in the erythroid compartment accounts for increased hemoglobin indicating a deregulated erythropoiesis.^4^

A number of patients with erythrocytosis in our cohort carried *TFR2* alterations, the most frequent being p.Arg752His, which is considered benign though at present little is known. Five other unknown variants have been observed and the quoted alterations were found alone or associated with variants on other genes (*EGLN1*, *JAK2*, *HFE*) known to be involved in erythrocytosis. To the best of our knowledge, this is the first paper describing the alteration of *TFR2* in patients with erythrocytosis, reinforcing the hypothesis of a relation between iron metabolism gene variants and erythrocytosis, mainly when associated with other gene variants.^5^

The present study brings additional support to the potential relationship between genes involved in iron metabolism and erythrocytosis and suggests that at least some cases are of a multigenic nature.^5^ Further studies are required to confirm our observations and the role played by the variants reported remains to be further elucidated.

## Author contributions

A.B. and M.L.R. conceived the study, and I.B. followed the patients.

## Conflict of interests

The authors have no disclosure to declare.

## Funding

This work was supported by DOR 2020 funding from the Department of Internal Medicine - DIMED, University of Padua, Padova, Italy.
