# Supplementary material for: TFR2 gene alterations in idiopathic erythrocytosis reinforce a possible relation between erythrocytosis and iron metabolism
Source: Genes Dis. 2024 Apr 5;12(1):101291. doi: 10.1016/j.gendis.2024.101291 (PMC11462191; doi:10.1016/j.gendis.2024.101291)
Supplement: Multimedia component 1 [file mmc1.docx]

**Table S1:** Main data of the 11 patients with *TFR2* variant, no clinical event occurred.

| Sex/Age | RBC  [x10^12^/L] | Hb [g/L] | HT  [%] | Ferritin [μg/L] | Serum EPO [IU/L] | Variant | Associated Variants |
| --- | --- | --- | --- | --- | --- | --- | --- |
| M/40 | 5.56 | 174 | 49.4 | 457 | 9.5 | *TFR2*R752H | none |
| M/59 | 5.98 | 171 | 49.8 | n.a. | 9.1 | *TFR2*R752H | none |
| M/20 | 5.36 | 166 | 54.8 | 74 | n.a. | *TFR2*R752H | none |
| M/39 | 6.06 | 177 | 50.9 | n.a. | 11.3 | *TFR2*R752H | none |
| M/70 | 5.8 | 175 | 53.6 | 150 | 5.6 | *TFR2*D602fs | none |
| M/58 | 5.4 | 172 | 50.7 | n.a. | n.a. | *TFR2*D127E | none |
| M/50 | 5.62 | 173 | 50.7 | n.a. | n.a. | *TFR2*R752H | *EGLN1*C127S |
| M/64 | 5.66 | 170 | 51 | 185 | 20 | T*FR2*D648Y | *EGLN1*Q157H |
| F/73 | 5.67 | 172 | 49 | 345 | 4.6 | *TFR2*A182E | J*AK2*L113V |
| M/31 | 5.59 | 173 | 49.5 | 191 | 6.6 | *TFR2*R752H | *HFE*H63D |
| M/69 | 7.13 | 183 | 59.1 | 559 | 13 | *TFR2*R752H | *JAK2*T78I *+*  *HFE*H63D/C282Y |

M = Male, F = Female, RBC = Red Blood Cells, Hb = Haemoglobin (normal range males 135g/L-165g/L and normal range females 120g/L-160g/L), HT = Haematocrit (normal range males 40%-49% and normal range females 37%-48%), EPO = Erythropoietin, n.a.= not available

**Table S2:** Prediction of *TFR2* variants

| Gene / Chr | Coding  Region  Change | Protein  Change | Exon | rs code | ACMG  Classification | ClinVar Prediction | gnomAD  Prediction | Mobidetails |
| --- | --- | --- | --- | --- | --- | --- | --- | --- |
| *TFR2*  Chr7 | c.381C>A | p.Asp127Glu | 3 | [rs145795884](https://www.ncbi.nlm.nih.gov/snp/rs145795884) | Likely Benign | Uncertain Significance | Uncertain Significance | Neutral |
|  | c.545C>A | p.Ala182Glu | 4 | / | Likely Benign | Unknown | Unknown | Intolerant |
|  | c.1804delG | p.Asp602fs | 16 | / | Uncertain Significance | Unknown | Unknown | / |
|  | c.1942G>T | p.Asp648Tyr | 16 | / | Uncertain Significance | Unknown | Unknown | / |
|  | c.2255G>A | p.Arg752His | 18 | [rs41295942](https://www.ncbi.nlm.nih.gov/snp/rs41295942) | Benign | Benign /  Likely Benign | Benign /  Likely Benign | Slightly tolerant |

## **MATERIAL AND METHODS**

## From 2013 to 2020, in our centre, we encountered 118 (M/F =101/17; mean age 53.7±17.2 years) patients who were diagnosed with idiopathic erythrocytosis. Patients’ inclusion criteria were: (i) haemoglobin >165 g/L and haematocrit >49% in males and haemoglobin >160 g/L and haematocrit >48% in females, (ii) long standing unexplained erythrocytosis, (iii) no relatives with increased Hb or HT, (iv) no evidence of smoke, arterial-venous shunt, pulmonary and/or renal diseases or neoplasms, (v) absence of somatic mutations in *JAK2* (p.Val617Phe or variants in *JAK2* exon 12). Patients carrying haemoglobin variants with high oxygen affinity, as evaluated by venous p50 and those with a left-shifted oxygen dissociation curve were excluded. No children younger than 16 years of age are included in the present cohort being our surgery dedicated only to adults.

The patients' data were collected in an ad hoc performed anonymous database approved by the Ethics Committee (EC) of the General Hospital-University of Padova, Italy. The patients gave consent to data collection for scientific purposes and publication. The study was conducted in accordance with the Declaration of Helsinki.

Thrombotic and haemorrhagic events were recorded. We collected red blood cells (RBC) counts, Hb and HT levels in all patients, ferritin levels in 67, transferrin saturation (T-Sat) in 38, and serum erythropoietin (EPO) in 66.

According to the manufacturer's instructions, DNA was extracted from granulocytes obtained from patients’ blood samples using EuroGold Blood DNA Mini Kit Plus (EuroClone) and stored at -20°C. DNA concentration was determined by Qubit 4 Fluorometer with Qubit^TM^ 1X dsDNA HS Assay Kits (Thermo Fisher Scientific).

A targeted NGS panel for patients with erythrocytosis was set up. The panel included the coding sequence of 14 genes (*BPGM, EGLN1, EPAS1, HAMP, FTL, HFE, HFE2, JAK2, SLC11A2, SLC40A1, TFR2, VHL, FTH1, EPOR*) involved in erythrocytosis because we choose to obtain a targeted sequence of genes interested in a specific group of patients. The panel is an “On Demand AmpliSeq Panel” designed with the Illumina Design Studio platform and validated in silico by Illumina. Libraries were prepared by using “AmpliSeq for Illumina On-Demand, Custom, and Community Panels” (Illumina) as per manufacturer’s instructions. The sequencing was performed in a MiSeq Illumina instrument (151 cycles paired end; mean target coverage 500X for each sample). FASTAQ data were analysed by Illumina BaseSpace softwares and by CLC Genomics Workbench Qiagen tools. *Homo sapiens* genome assembly GRCh37 (hg19) was used as reference and Integrative Genomics Viewer (IGV) to visualize reads alignment. Germline and somatic variants were filtered using two independent and different workflows primarily based on different frequency thresholds for variant identification. In both workflows, the selection of variants was carried out through multiple filters that mainly consider the total coverage of the fragment of interest, the quality and the correct and minimum number of base calls. Only non-synonymous variants that passed all filters were considered for further validation and analysis. Germline variants identified by NGS were confirmed by direct Sanger sequencing.

Amplification conditions and primers are available upon request. The clinical significance of the identified variants was inferred using ClinVar, OMIM database, MobiDetails, gnomAD and according to ACMG guidelines with the help of VARSOME.
